# Occurrence of Idiopathic Pulmonary Fibrosis in Italy: Latest Evidence from Real-World Data

**DOI:** 10.3390/ijerph19052510

**Published:** 2022-02-22

**Authors:** Marica Iommi, Martina Bonifazi, Andrea Faragalli, Lara Letizia Latini, Federico Mei, Liana Spazzafumo, Edlira Skrami, Luigi Ferrante, Flavia Carle, Rosaria Gesuita

**Affiliations:** 1 Center of Epidemiology, Biostatistics and Medical Information Technology, Department of Biomedical Sciences and Public Health, Marche Polytechnic University, 60126 Ancona, Italy; m.iommi@staff.univpm.it (M.I.); a.faragalli@staff.univpm.it (A.F.); e.skrami@staff.univpm.it (E.S.); l.ferrante@staff.univpm.it (L.F.); r.gesuita@staff.univpm.it (R.G.); 2 Department of Biomedical Sciences and Public Health, Marche Polytechnic University, 60126 Ancona, Italy; m.bonifazi@staff.univpm.it (M.B.); letizia.latini@outlook.it (L.L.L.); f.mei@staff.univpm.it (F.M.); 3 Respiratory Diseases Unit, Azienda Ospedaliero-Universitaria “Ospedali Riuniti”, 60126 Ancona, Italy; 4 Regional Health Agency of Marche, 60125 Ancona, Italy; liana.spazzafumo@regione.marche.it; 5 National Centre for Healthcare Research and Pharmacoepidemiology, 20126 Milano, Italy

**Keywords:** idiopathic pulmonary fibrosis, incidence, temporal trend, real-world data, clinical epidemiology

## Abstract

The aim of the study was to evaluate the trend in the incidence of idiopathic pulmonary fibrosis (IPF) in a real-world setting of the Marche region, a region of Central Italy, between 2014 and 2019. This observational prospective study was based on administrative databases of hospital discharges and drug prescriptions. All adult residents in the Marche Region with a first prescription of antifibrotic drugs, or a first hospitalization with a diagnosis of IPF during the study period, were identified as incident cases of IPF. A multiple Poisson regression analysis was used to estimate the IPF incidence trend, adjusted for age, sex, and health conditions. The mean incidence rate was 9.8 cases per 100,000 person-years. A significant increasing trend of 6% per year was observed. The incidence rates were significantly higher in males than females, older subjects, and those with poorer health conditions. To our knowledge, this is the first study evaluating incidences of IPF over a 6-year period in Italy, combining hospital discharge and drug prescription databases. The study highlights that the combined use of two secondary sources is a reliable strategy to accurately identify new cases of IPF when the appropriate disease registry is lacking.

## 1. Introduction

Idiopathic pulmonary fibrosis (IPF) is a rare, chronic, and progressive disease, causing an irreversible decline of lung function over time that dramatically impairs the quality of life. It mainly occurs in older male people, and it is characterized by a poor prognosis, with a median survival time ranging between 3 and 5 years [1]. Aetiology has yet to be clearly established, although risk factors such as aging, smoking habits, occupational exposure, and air pollution, are likely to play a relevant role in IPF onset and progression in a predisposing genetic background [2,3].

Epidemiological estimates showed huge geographical variability [1,4], with the highest incidence rates in North America and East Europe (between 3 and 9 new cases per 100,000 person-years) and the lowest in East Asia (less than 4 new cases per 100,000 person-years) [5]. IPF incidence is increasing worldwide [5], as well as hospitalization [6] and mortality [7], indicating an increasing burden of the disease on the population and health systems.

Reasons for worldwide variability in IPF incidence and prevalence include potential differences in genetics and risk factor exposure among countries, as well as methodological issues in case detection, due to lack of uniform definition of IPF in older studies, differences in diagnostic criteria, classification terms, target population, study design, data sources, and data collection methods [4,8,9]. 

Most of the latest papers used administrative healthcare databases for an epidemiologic assessment, with the advantage of including very large populations (hospital discharges, outpatients visits, primary care databases, insurance databases, the Health Improvement Network death certificates), but with the potential risk of being less accurate in case identification, due to the absence of specific diagnosis codes [9,10,11,12,13,14]. The ICD-9-CM code 516.3, in fact, referring generically to “idiopathic interstitial pneumonia”, may lead to erroneously including other idiopathic non-IPF parenchymal lung diseases, such as nonspecific interstitial pneumonia or acute interstitial pneumonia. As a result, several algorithms have been proposed that use additional codes (related to diagnostic procedures and/or alternative diagnoses) and secondary sources to improve the case identification procedure [8,15]. 

Since the approval in clinical practice of antifibrotic drugs (Pirfenidone and Nintedanib) for treatment of mild to moderate IPF, in 2014 and in 2016 (in Europe), respectively, drug prescription healthcare databases can be used in the identification of prevalent and newly diagnosed cases.

The epidemiology of IPF in Italy has been recently assessed in a few studies at the regional level (Lombardy and Lazio) based on regional hospital discharge databases [8,16] or primary care databases [9]. In the present study, we aimed to assess the overall prevalence and incidence, and the annual incidence of IPF in an Italian region of Central Italy, the Marche Region, between 2014 and 2019, using two regional healthcare sources, hospital discharge, and drug prescriptions databases.

## 2. Materials and Methods

### 2.1. Study Design, Population, and Data Sources

In this observational longitudinal prospective study, the target population included all adult beneficiaries of the National Health Service (NHS) resident in the Marche Region, a region of Central Italy, with about 1.5 million inhabitants. The study period was between 2014 and 2019.

Demographic, socio-economic, and health characteristics between the populations of the Marche region and Italy were compared using the indicators calculated in 2019 by the Italian National Institute of Statistics (ISTAT) [17].

IPF prevalent and incident cases were retrieved using healthcare administrative databases of the Health Regional System: (1) Regional Beneficiaries database (RBD) which includes the identification code of the beneficiary, date of birth, date of death, sex, assistance start, and end dates; (2) Hospital Discharges (HD) database reporting information on admission and discharge dates, primary and up to five secondary diagnoses, up to six interventions (coded using International Classification of Diseases, 9th Revision Clinical Modification, ICD-9-CM); (3) Drug prescriptions (DP) database which contains drug prescriptions (coded according to the Anatomical Therapeutic Chemical, ATC classification system) reimbursed by the Nation Health Service (NHS); (4) Outpatient care database (OCD) which reports outpatient specialistic visits and outpatient exams reimbursed by the NHS. The four databases were linked using a deterministic procedure based on the beneficiary’s identification code.

### 2.2. IPF Case Identification Algorithm

Two secondary sources were used in the IPF case identification algorithm, HD, and DP databases.

Patients having at least one drug prescription of Pirfenidone (ATC: L04AX05) or Nintedanib (ATC: L01XE31–L01EX09), in the study period, were identified as IPF cases.

Using the HD database, IPF cases were identified according to the three definitions previously proposed in the literature [8,18] which are based on progressively tighter criteria: (1) General Case Definition (GCD), all individuals with at least one hospitalization with a diagnosis of IPF (ICD-9-CM code 516.3); (2) Broad Case Definition (BCD), patients that satisfied the GCD and had no hospitalization with a diagnosis code for any other type of ILDs; (3) Narrow Case Definition (NCD), patients who met BCD criteria and had at least an inpatient procedure code for surgical lung biopsy (ICD-9-CM 33.28, 34.21), transbronchial lung biopsy (ICD-9-CM 33.27), or an inpatient/outpatient code for computed tomography of the thorax (ICD-9-CM: 87.41) within one month before the hospitalization for IPF (Table S1).

Therefore, IPF cases were considered incidents if their first hospitalization, according to the three case definition algorithms, or first drug prescription occurred during 2014–2019. The date of the first health service contact for IPF was used as a proxy of disease diagnosis (index date). Cases residing for less than 3 years before the index date in the Marche region or with hospitalization for IPF or antifibrotic prescription in 2011–2013 were excluded. Patients treated with Nintedanib only in 2014–2019 who had been diagnosed with lung cancer in the 3 years prior to the index date were excluded.

### 2.3. Accuracy of Secondary Sources in Identifying New Cases of IPF

A comparison with confirmed IPF cases from the Respiratory Disease Unit, “Azienda Ospedaliero-Universitaria Ospedali Riuniti” of Ancona, was carried out to assess the accuracy of the HD database and DP in identifying the IPF cases, reviewing the clinical charts of 2014–2019 (M.B., L.L.). This hospital is the reference hospital for the Marche region and accounted for 25% of all the 584 first hospital admissions and 27% of the 1055 hospitalizations with the diagnosis of IPF in the study period.

Confirmed IPF cases were then matched with IPF cases detected by the two secondary sources, using the beneficiary’s identification code, by one of the co-authors (M.B.) according to the Italian Personal Data Protection Law. In the comparison, the three case definitions were used, and the proportion of incident cases detected by the secondary sources, the true positive rate, on the total cases identified by the clinical charts, was estimated.

### 2.4. Statistical Analysis

Continuous variables were summarized by mean values and standard deviations or median values and interquartile range (IQR), according to the shape of data distribution. Absolute and percentage frequencies were used to summarize qualitative variables.

The mean 2014–2019 IPF annual prevalence rate per 100,000 residents and 95% confidence interval (95% CI), and the mean and per year IPF incidence rates per 100,000 person-years (py), were estimated by adding the cases identified by the two sources on the total population of the Marche region aged ≥18 years on 1 January of each year between 2014–2019 reported by ISTAT [17].

IPF incidence rates were analysed by sex, age groups (18–54, 55–75, ≥75 years) at the disease diagnosis, and health conditions measured by the Multisource Comorbidity Score (MCS) [19]. The MCS evaluated patients’ health status in the two years preceding the index date and was considered in classes (0–4, 5–14, ≥15, higher scores indicate worse health conditions).

A Poisson regression analysis was used to evaluate the overall incidence trend and stratified by age and sex. In presence of an excess of zero values, a zero-inflated Poisson model was used. The annual trend in IPF incidence was estimated using multiple Poisson regression analyses, adjusted for age, sex, and MCS classes. The proportion of residents in each MCS class, by age and sex, was estimated using the database of beneficiaries of the Marche region in 2017. These proportions were applied to the target population in each year of the study period to obtain the offset to be used in the Poisson model. For each Poisson model, the null hypothesis of equidispersion was tested against the alternative of overdispersion [20].

The significance level for all the analyses was set at *p* < 0.05. Statistical analyses were performed using R, version 4.1.0.

## 3. Results

### 3.1. Accuracy of Secondary Sources in Detecting New IPF Cases

One-hundred and forty-two new IPF cases with confirmed diagnosis during 2014–2019 were identified by the clinical charts of the Respiratory Disease Unit of Ancona.

The GCD algorithm correctly tracked 121 out of 142 subjects with IPF, with a true positive rate of 85.2% (Figure S1). Among the patients from the clinical charts of the Respiratory Disease Unit, 10 were not tracked by the GCD algorithm because 8 subjects (5.6%) had had a hospitalization before 2014 and 2 (1.4%) had not met the inclusion criteria of residence. In total, 11 subjects (7.7%) were never tracked by the secondary sources. Considering the BCD algorithm, the true positive rate dropped to 64.8% (92 out of 142), and with the NCD algorithm decreased to 58.5% (83 out of 142).

Table 1 shows the number of cases and the incidence rates with 95% CI of IPF according to case definitions. Compared to the number of new cases detected by the GCD algorithm, 147 and 238 patients were excluded using BCD and NCD algorithms respectively. Consequently, the overall incidence rate drops to 8.1 (95% CI: 7.5; 8.7) with the BCD algorithm and to 6.9 (95% CI: 6.3; 7.5) with the NCD algorithm.

The incidence rate of IPF in the study period ranged between 7.6 and 12.9 per 100,000 py using the GCD algorithm, between 5.8 and 10.7 per 100,000 py using the BCD algorithm, and from 4.4 to 9.3 per 100,000 py using the NCD algorithm. A significant increasing trend over time was observed for all case definitions (*p* < 0.001). No significant overdispersion was observed in each case definition.

Considering the high true positive rate of the GCD algorithm and the dramatic reduction of the true positive rate using the BCD algorithm and the NCD algorithm, we decided to perform all the analyses using the GCD algorithm.

### 3.2. IPF Incidence Rate

Between 2014 and 2019, 1037 prevalent and 766 new IPF cases were found using the GCD algorithm, with an overall prevalence of 13.3 (95% CI: 12.5; 14.1) per 100,000 residents and a mean incidence rate of 9.8 (95% CI: 9.1; 10.6) per 100,000 py.

Of the 766 new cases of IPF, 521 (68.0%) were males, with a median age of 75 years (IQR: 68.3–80); 145 (18.9%), 462 (60.3%), 37 (4.8%), and 122 (15.9%) subjects were identified only by DP, only by HD, first by DP then by HD, first by HD then by DP databases, respectively. The number of new cases detected only by DP source increased during the study period from 5 out of 114 in 2014 to 50 out of 138 in 2019.

The median MCS class was 5–14, 319 (41.6%) subjects had an MCS between 0 and 4, and 108 (14.1%) had a score ≥15.

Table 2 shows IPF incidence cases, person-years, and rates over the entire period by sex, age classes, and health condition measured by MCS classes. Males had an overall incidence rate of 14.0 (95% CI: 12.8; 15.2) per 100,000 py, 2.3 (95% CI: 2.0; 2.7) fold higher than females (6.0; 95% CI: 5.3; 6.8). IPF incidence increased with age both for males and females; rates under 55 years ranged between 0.5 and 1.6 per 100,000 py, between 6.0 and 25.3 per 100,000 py in the age group 55–74, and between 12.4 and 82.6 per 100,000 py in subjects aged 75 years or more (Table S2).

The incidence rate in MCS class 5–14 was 7.6 (95% CI: 6.6; 8.9) fold higher than class 0–4, and MCS class ≥15 was 1.4 (95% CI: 1.1; 1.7) fold higher compared to class 5–14 (Table 2).

### 3.3. Trend Analysis

**Figure 1:**
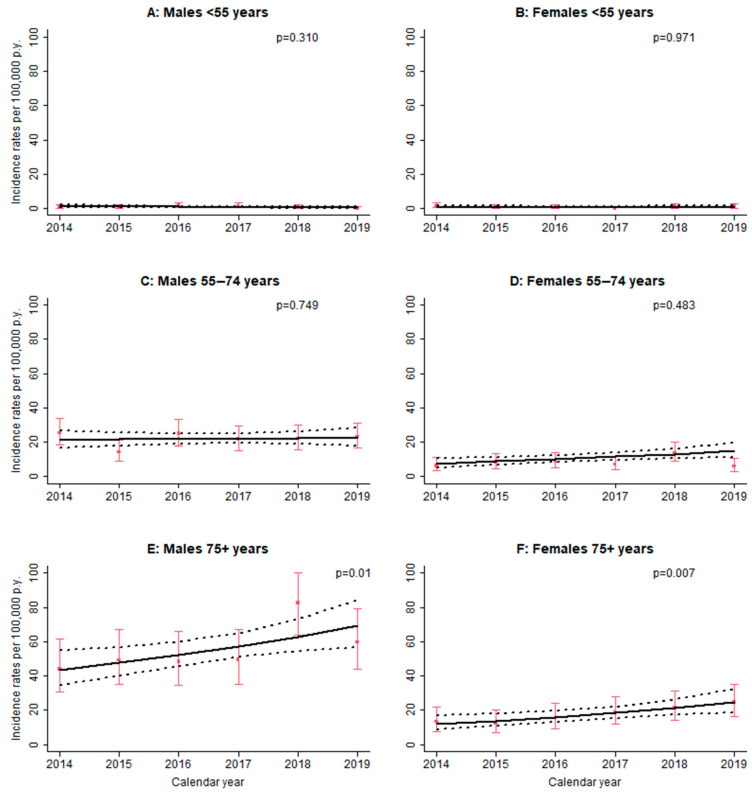
Observed (red squares) and estimated (solid line) annual IPF incidence trend by age and sex (red bars: 95% CI of observed incidence rate; dot lines: 95% confidence band of estimated incidence trend; *p*-values refer to annual trend).

Figure 1 shows the annual IPF incidence trend by age and sex. The Poisson regression model was used for all the strata, as only two zero values in two different strata (males < 55 years in 2019 and females < 55 years in 2017) were observed. A significant increasing trend over time was observed in age ≥75 both for males and females, with an annual increase of 10% (95% CI: 2.2%; 17.8%) and 15% (95% CI: 4.0%; 27.8%), respectively. No significant overdispersion was observed in each age and sex strata.

Table 3 shows the results of the multiple Poisson regression analysis. A significant increasing trend of 6% per year was observed in the study period. The incidence rates were significantly higher in males than females, in older subjects (55–74 and ≥75 years) and in those with worse health conditions (MCS 5–14 and ≥15).

### 3.4. Comparison between the Populations of Marche Region and Italy

Table S3 and Figure S2 show the comparison of demographic, socio-economic, and health indicators between the Marche region and Italian populations in 2019. The two populations showed a similar profile, with an overlapping age and sex structure (Figure S2) and a small difference in the birth and death rate (0.6‰ and −0.9‰, respectively), the mean number of children per woman, and life expectancy at birth (−0.8 years); the unemployed rate and the individual relative poverty rate were slightly lower in Marche region (5.0 vs. 4.5% and 14.7 vs. 13.2, respectively), while the educational level was basically the same. Regarding the health status, negligible differences were observed between Italy and the Marche region in terms of people in good health (1.2%), people with at least one chronic condition (−0.6%), percentage of obese people (−0.7%), percentage of smokers (2.3%), and people with at least one hospitalization (−0.3‰) (Table S3).

## 4. Discussion

To our knowledge, this is the first study providing epidemiologic estimates of IPF over a 6-year period, using both the hospital discharge and the drug prescription databases, in an Italian region of approximately 1.5 million inhabitants, which can be considered representative of the Italian population from a demographic, socio-economic point of view, and for the distribution of the main health indicators. The estimated mean of the annual incidence rates of IPF ranged between a minimum of 7.6 in 2015 and a maximum of 12.8 in 2018 per 100,000 person-years over the study period. These findings confirm that Italy ranks as one of the countries with the highest incidence rates in Europe [21], as already shown by other Italian studies [8,16] conducted at the regional level and based on the GCD. However, the rates reported by these Italian studies (5.3 and 7.5 per 100,000 py, respectively) were slightly lower than ours, probably because they referred to a previous study period (2005–2010 and 2005–2009, respectively) and were based only on the hospital discharge database. A recent Italian population-based study [9], despite using the ICD-9 CM code 516.3 to identify IPF new cases, reported lower incidence rates, ranging between 2.2 and 3.8 per 100,000 py for the period 2014–2017 in Lombardy. This sizable difference may be due to the different secondary source used in the study, the Health Search Database, employed in the Italian primary care setting.

Our study also revealed a significant temporal trend in IPF incidence rates of 6% over the period 2014–2019 (Table 3), more evident in the oldest age group (Figure 1). Few studies have evaluated the incidence trend, most of which reported a positive increase. A significant trend was observed in two UK studies, reporting an annual increase of 5% between 1998 and 2010 [6] and of 78% in 2012 compared with the year 2000 [11]; in Spain [12], the incidence significantly increased from 3.82 per 100,000 in 2004 to 6.98 in 2013; in Sweden, the incidence of IPF ranged from 10.4 to 15.4 cases per 100,000 population per year between 2001 and 2015 [22]. Two studies conducted in Taiwan [10,23] using both broad and narrow case definitions, reported yearly increasing incidence rates during 2001–2011 and 1997–2007, respectively. Conversely, two studies reported a stable or slight decrease trend [8,24] over time. However, Harari et al. [8] observed a significant drop in incidence rate at the end of the study period (2009) explained by the change in legislation establishing administrative and management protocols that induced a temporary reduction in the number of hospitalizations. In a subsequent paper, Harari et al. [9] reported that the incidence rate started to increase in the last 3 years of the study period (2002–2017), probably due to a growing awareness for IPF disease.

The widespread trend of increasing IPF incidence in recent years is likely the result of a reduction in undiagnosed cases, due to an improved awareness among physicians through the development of educational programs, and a real increase in cases, due to the progressive aging of the overall population and the worsening of air pollution [25].

The GCD algorithm, based on the ICD-9 CM 516.3 code applied to the hospital discharge database, showed the highest rate of true positives in evaluating the accuracy of secondary sources. Although the diagnosis of IPF remains a complex procedure involving a multidisciplinary team of specialists, the diagnostic criteria have been continuously refined especially since 2011, providing increasingly accurate recommendations, and lowering IPF misdiagnosis. In addition, new specific treatments for IPF have been introduced in Italy since 2014, so the drug prescriptions database represents a reliable secondary source in the process of IPF case identification. In our study, a remarkable amount of new IPF cases would have been lost (145 out 766) if the drug prescription database had not been considered, whereas 37 would have been detected later by hospitalization; in fact, therapy is indicated in patients with mild/moderate IPF, who can be diagnosed and managed outpatient and who may not be hospitalized in the short term. Therefore, the combined use of these two secondary sources allowed us to estimate the incidence rates over time more accurately.

This study has some limitations, related to the type of data sources used that have administrative purposes and are not originally designed to be used in epidemiological studies. Some degree of inaccuracy as to the actual timing of the first IPF diagnosis needs to be considered: new cases are identified through their first IPF hospitalization or their first antifibrotic prescription, both of which may, however, occur several months after an outpatient diagnosis. Furthermore, the procedure used to evaluate the accuracy of the three algorithms used to detect IPF cases from secondary sources was able to estimate the rate of true positives, but not one of the false positives, thus an unknown number of cases of false IPFs may have been included in the study. Patients who did not receive antifibrotics due to the absence of eligibility criteria for therapy and who were not hospitalized during the study period cannot be identified.

Another limitation is that the Marche region is a small region of Central Italy, which constitutes about 2.5% of the Italian population; however, it should be emphasized that secondary sources include all the resident beneficiaries of NHS and their access to the healthcare system. In addition, the design of the study and the use of the prescription database in addition to the hospital discharge database allows us to accurately identify the clinical history of each resident. Finally, the Marche region shows demographic, socio-economic, and health profiles similar to those observed for the Italian population. In fact, no significant differences were observed between the Marche region and Italian population in terms of demographic and socio-economic parameters, and for the distribution of main health indicators; therefore, our results can give a picture of the distribution of the IPF disease nationwide.

## 5. Conclusions

In conclusion, the incidence and temporal trend of IPF observed in our study are consistent with the results of previous Italian and European studies. Moreover, the study highlights that the combined use of hospital discharge and pharmaceutical prescriptions is a reliable strategy to accurately identify new cases of IPF.

## Figures and Tables

**Table 1:** Incidence rate per 100,000 py over the 2014–2019 period by case definition.

	Total 2014–2019	2014	2015	2016	2017	2018	2019	*p*
**Source of identification**								
Only DP	145 (18.9%)	5 (4.4%)	7 (7.1%)	18 (14.1%)	22 (18.2%)	43 (25.9%)	50 (36.2%)	
Only HD	462 (60.3%)	88 (77.2%)	78 (78.8%)	77 (60.2%)	70 (57.9%)	76 (45.8%)	73 (52.9%)	
1° DP, 2° HD	37 (4.8%)	6 (5.3%)	6 (6.1%)	7 (5.5%)	6 (5%)	10 (6%)	2 (1.4%)	
1° HD, 2° DP	122 (15.9%)	15 (13.2%)	8 (8.1%)	26 (20.3%)	23 (19%)	37 (22.3%)	13 (9.4%)	
**Cases based on DP or GCD algorithm**	766	114	99	128	121	166	138	<0.001 ^a^
Incidence rate	9.8	8.7	7.6	9.8	9.3	12.8	10.7	
(95% IC)	(9.1; 10.6)	(7.2; 10.5)	(6.2; 9.2)	(8.2; 11.7)	(7.7; 11.1)	(10.9; 14.9)	(9.0; 12.7)	
**Cases based on DP or BCD algorithm**	619	90	76	100	98	137	118	<0.001 ^b^
Incidence rate	7.9	6.9	5.8	7.7	7.6	10.6	9.2	
(95% IC)	(7.3; 8.6)	(5.5; 8.5)	(4.6; 7.3)	(6.3; 9.4)	(6.1; 9.2)	(8.9; 12.5)	(7.6; 11.0)	
**Cases based on DP or NCD algorithm**	528	71	58	88	87	120	104	<0.001 ^c^
Incidence rate	6.8	5.4	4.4	6.8	6.7	9.3	8.1	
(95% IC)	(6.2; 7.4)	(4.2; 6.9)	(3.4; 5.7)	(5.4; 8.3)	(5.4; 8.3)	(7.7; 11.1)	(6.6; 9.8)	
py	7,789,720	1,305,394	1,304,422	1,300,347	1,297,513	1,294,161	1,287,883	

**DP**: Drug Prescription database. **HD**: Hospital Discharge database. **GCD**: General Case Definition, all individuals with at least one hospitalization with a diagnosis of IPF (ICD-9-CM code 516.3); **BCD**: Broad Case Definition, patients that satisfied the GCD and had no hospitalization with a diagnosis code for any other type of ILDs; **NCD**: Narrow case Definition, patients who met BCD criteria and had at least an inpatient procedure code for surgical lung biopsy (ICD-9-CM 33.28, 34.21), or transbronchial lung biopsy (ICD-9-CM 33.27), or an inpatient/outpatient code for computed tomography of the thorax (ICD-9-CM: 87.41) within one month before the hospitalization for IPF. **py**: Total resident population ≥ 18 years, January 1st. ***p***: *p*-value from Poisson regression analysis of the trend. Overdispersion test results: (z = 0.881, *p* = 0.189) ^a^; (z = 0.625, *p* = 0.266) ^b^; (z = 0.731, *p* = 0.232) ^c^.

**Table 2:** New cases of IPF and incidence rate per 100,000 py (95% CI) by sex, age, and health condition (MCS classes), over the 2014–2019 period.

	Cases	Person Years (py)	Incidence Rate per 100,000 py (95% CI)
**Total**	766	7,789,720	9.8 (9.1; 10.6)
**Males**			
*<55 years*	18	2,177,922	0.8 (0.5; 1.3)
*55–74 years*	235	1,073,062	21.9 (19.2; 24.9)
*≥75 years*	268	481,232	55.7 (49.2; 62.8)
*Total*	521	3,732,216	14 (12.8; 15.2)
**Females**			
*<55 years*	18	2,164,029	0.8 (0.5; 1.3)
*55–74 years*	98	1,165,956	8.4 (6.8; 10.2)
*≥75 years*	129	727,519	17.7 (14.8; 21.1)
*Total*	245	4,057,504	6 (5.3; 6.8)
**MCS classes**			
*MCS 0–4*	319	6,648,674	4.8 (4.3; 5.4)
*MCS 5–14*	339	924,602	36.7 (32.9; 40.8)
*MCS ≥15*	108	216,444	49.9 (40.9; 60.2)

95% CI: 95% confidence interval. MCS: Multisource Comorbidity Score.

**Table 3:** Annual trend of IPF incidence adjusted for age, sex, and patients’ health condition at diagnosis. Results of multiple Poisson regression models.

	IRR	95% CI	*p*-value
**Calendar year**	1.06	1.02; 1.11	0.004
**Gender**			
Male vs. Female	2.70	2.32; 3.15	<0.001
**Age groups (years)**			
55–74 vs. <55	13.26	9.51; 19.08	<0.001
≥75 vs. <55	22.37	15.95; 32.32	<0.001
**MCS**			
MCS 5–14 vs. 0–4	4.05	3.46; 4.75	<0.001
MCS ≥15 vs. 0–4	4.67	3.71; 5.83	<0.001

**IRR**: incidence rate ratio. **95% CI**: 95% confidence interval. **MCS**: Multisource comorbidity score. Overdispersion test result: (z = 1.284, *p* = 0.100).

## Data Availability

Restrictions apply to the availability of these data. Data were obtained from the Marche region and are available with the permission of the Marche region.

## Supplementary Materials

The following supporting information can be downloaded at: https://www.mdpi.com/article/10.3390/ijerph19052510/s1, Table S1: Criteria applied in general, broad, and narrow case definitions for IPF. Figure S1: Accuracy of secondary sources in detecting new IPF cases. Procedure and results. Table S2: Incidence cases and rates (100,000 person-years) of IPF between 2014–2019 in the Marche region by age groups and sex. Table S3: Comparison of demographic, socio-economic, and health characteristics between the Marche population and the Italian population. Figure S2: Age and sex population pyramid of the Marche and Italy populations of 2019.
