# Supplementary material for: Occurrence of Idiopathic Pulmonary Fibrosis in Italy: Latest Evidence from Real-World Data
Source: Int J Environ Res Public Health. 2022 Feb 22;19(5):2510. doi: 10.3390/ijerph19052510 (PMC8909740; doi:10.3390/ijerph19052510)
Supplement: Supplementary file 1 [file ijerph-19-02510-s001.zip › ijerph-1543050-supplementary.pdf]

Table S1 – Criteria applied in general, broad and narrow case definitions for IPF.

Algorithm	Criteria
General case definition (GCD)	Individuals with at least one hospitalization with diagnosis of IPF (ICD-9-CM code 516.3)
Broad case definition (BCD)	Individuals that satisfied the GCD and had no hospitalization with a diagnosis code for any other type of ILDs (ICD-9-CM codes 135, 272.7, 277.3, 277.8, 446.21, 446.4, 495, 500, 501, 502, 503, 504, 505, 506.4, 508.1, 508.8, 515, 516.0, 516.1, 516.2, 516.8, 516.9, 517.2, 517.8, 518.3, 555, 710.0, 710.1, 710.2, 710.3, 710.4, 714.81, 720.0, 759.5) on or after date of last IPF diagnosis (ICD-9-CM code 516.3)
Narrow case definition (NCD)	Individuals who met BCD criteria and had at least one of the following procedures within one month before the hospitalization for IPF:<br>- an inpatient procedure code for surgical lung biopsy (ICD-9-CM 33.28, 34.21), OR<br>- an inpatient procedure code for transbronchial lung biopsy (ICD-9-CM 33.27), OR<br>- an inpatient/outpatient code for computed tomography of the thorax (ICD-9-CM: 87.41)

Figure S1 – Accuracy of secondary sources in detecting new IPF cases. Procedure and results.

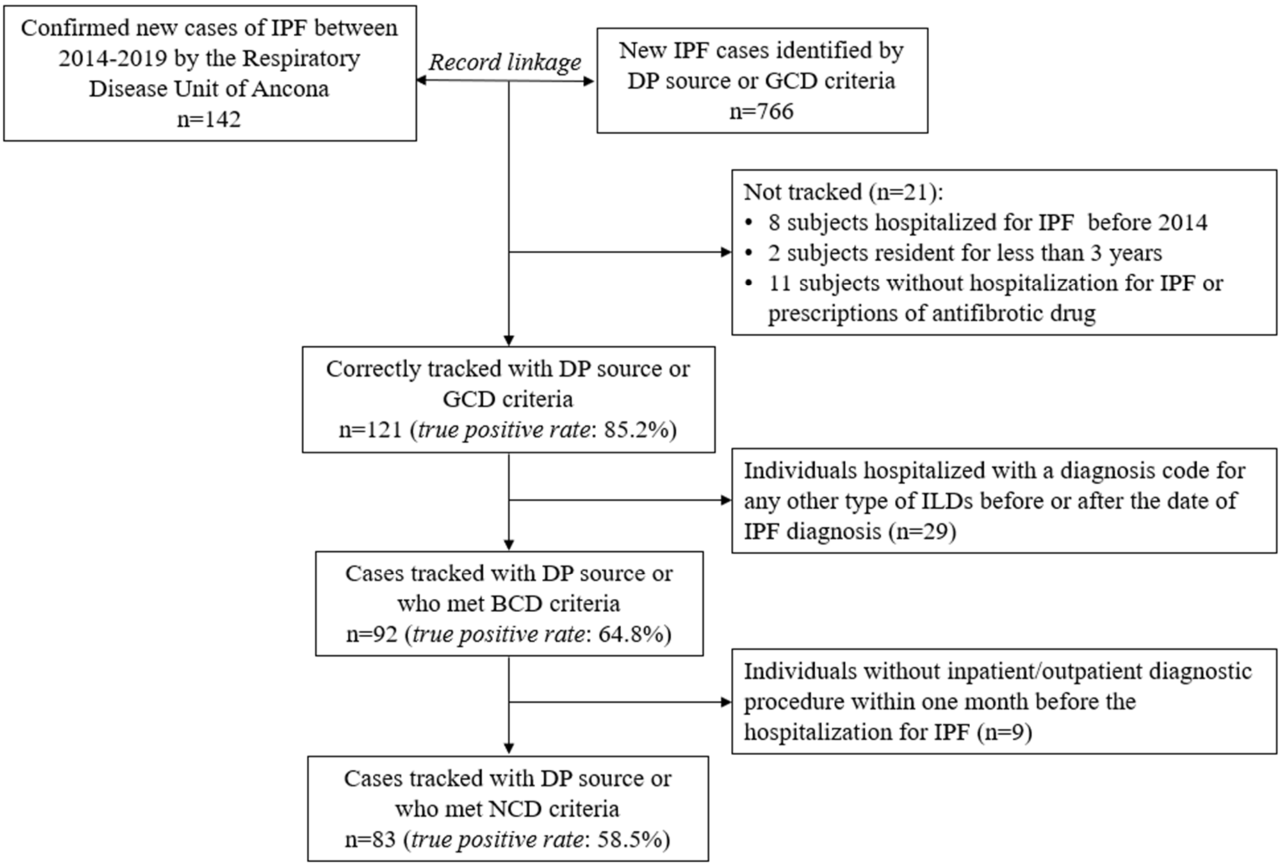

Table S2 – Incidence cases and rates (100,000 person-years) of IPF between 2014-2019 in Marche region by age groups and sex.

	2014		2015		2016		2017		2018		2019	
	IPF cases	IR per 100,000 py (95% CI)	IPF cases	IR per 100,000 py (95% CI)	IPF cases	IR per 100,000 py (95% CI)	IPF cases	IR per 100,000 py (95% CI)	IPF cases	IR per 100,000 py (95% CI)	IPF cases	IR per 100,000 py (95% CI)
**Male**	81	13 (10.3; 16.1)	66	10.6 (8.2; 13.5)	89	14.3 (11.5; 17.6)	84	13.5 (10.8; 16.7)	109	17.5 (14.4; 21.2)	92	14.9 (12; 18.2)
<55 years	3	0.8 (0.2; 2.3)	2	0.5 (0.1; 2)	6	1.6 (0.6; 3.6)	5	1.4 (0.4; 3.2)	2	0.6 (0.1; 2)	0	0 (0; 1)
55-74 years	44	25.3 (18.4; 34)	25	14.3 (9.2; 21.1)	44	24.9 (18.1; 33.5)	39	21.7 (15.4; 29.7)	40	21.9 (15.7; 29.8)	43	23.2 (16.8; 31.2)
≥75 years	34	44 (30.5; 61.6)	39	49.2 (35; 67.2)	39	48.4 (34.4; 66.2)	40	49.4 (35.3; 67.2)	67	82.6 (64; 104.9)	49	59.8 (44.2; 79)
**Female**	33	4.8 (3.3; 6.8)	33	4.8 (3.3; 6.8)	39	5.8 (4.1; 7.9)	37	5.5 (3.9; 7.5)	57	8.5 (6.4; 11)	46	6.9 (5; 9.2)
<55 years	5	1.3 (0.4; 3.1)	2	0.5 (0.1; 2)	3	0.8 (0.2; 2.4)	0	0 (0; 1)	4	1.1 (0.3; 2.9)	4	1.2 (0.3; 3)
55-74 years	12	6.3 (3.3; 11)	16	8.4 (4.8; 13.6)	17	8.8 (5.2; 14.2)	14	7.2 (3.9; 12)	27	13.7 (9; 19.9)	12	6 (3.1; 10.5)
≥75 years	16	13.5 (7.7; 21.9)	15	12.4 (6.9; 20.4)	19	15.6 (9.4; 24.3)	23	18.8 (11.9; 28.2)	26	21.4 (14; 31.4)	30	24.7 (16.6; 35.2)

IR: incidence rate; py: person-years

Table S3 – Comparison of demographic, socio-economic and health characteristics between the Marche population and the Italian population.

**Population characteristics**	**Italy**	**Marche**
**Demography**		
Birth rate (‰)	7.0	6.4
Death rate (‰)	10.6	11.5
Mean number of children per woman	1.3	1.2
Life expectancy at birth	83.2	84.0
Life expectancy at 65 years	21.0	21.6
Life expectancy at 85 years	6.7	6.8
**Socio-economic#**		
Employed rate (%)[a]	44.9	48.1
Unemployed rate (%)[b]	5.0	4.5
Individual relative poverty rate (%)[c]	14.7	13.2
Mean annual household income (€)	31,641	33,275
Education (%)[d]		
Low (below upper secondary)	48.6	47.5
Medium (upper secondary)	36.4	36.5
High (tertiary)	15.0	16.1
**Health Status#**		
People in good health (%)	68.8	67.6
People with at least one chronic condition (%)	40.9	41.5
People with at least two chronic conditions (%)	21.1	22.0
Body Mass Index (%)		
Underweight	3.0	2.9
Normal weight	50.6	51.1
Overweight	35.4	34.4
Obese	10.9	11.6
Smoke habit (%)[e]		
Smokers	18.4	16.1
Former smokers	23.0	24.7
Non-smokers	57.4	57.6
Mean number of cigarettes per day	11.1	11.2
Hospitalization[f]		
People with at least one hospitalization (‰)	29.4	29.7
Number of hospitalizations (‰)	33.4	36.2

#Sample estimates

a: % of employed on the population aged ≥15 years

b: % of unemployed on the population aged ≥15 years

c: % of people living in households in relative poverty over residents

d: % on the population aged ≥15 years

e: % on the population aged ≥14 years

f: in the 3 months prior to the interview

Note. Table adapted from "I.Stat – Il tuo accesso diretto alla statistica italiana", by the Italian National Institute of Statistics (ISTAT).

Figure S2 – Age and sex population pyramid of the Marche and Italy populations of 2019.

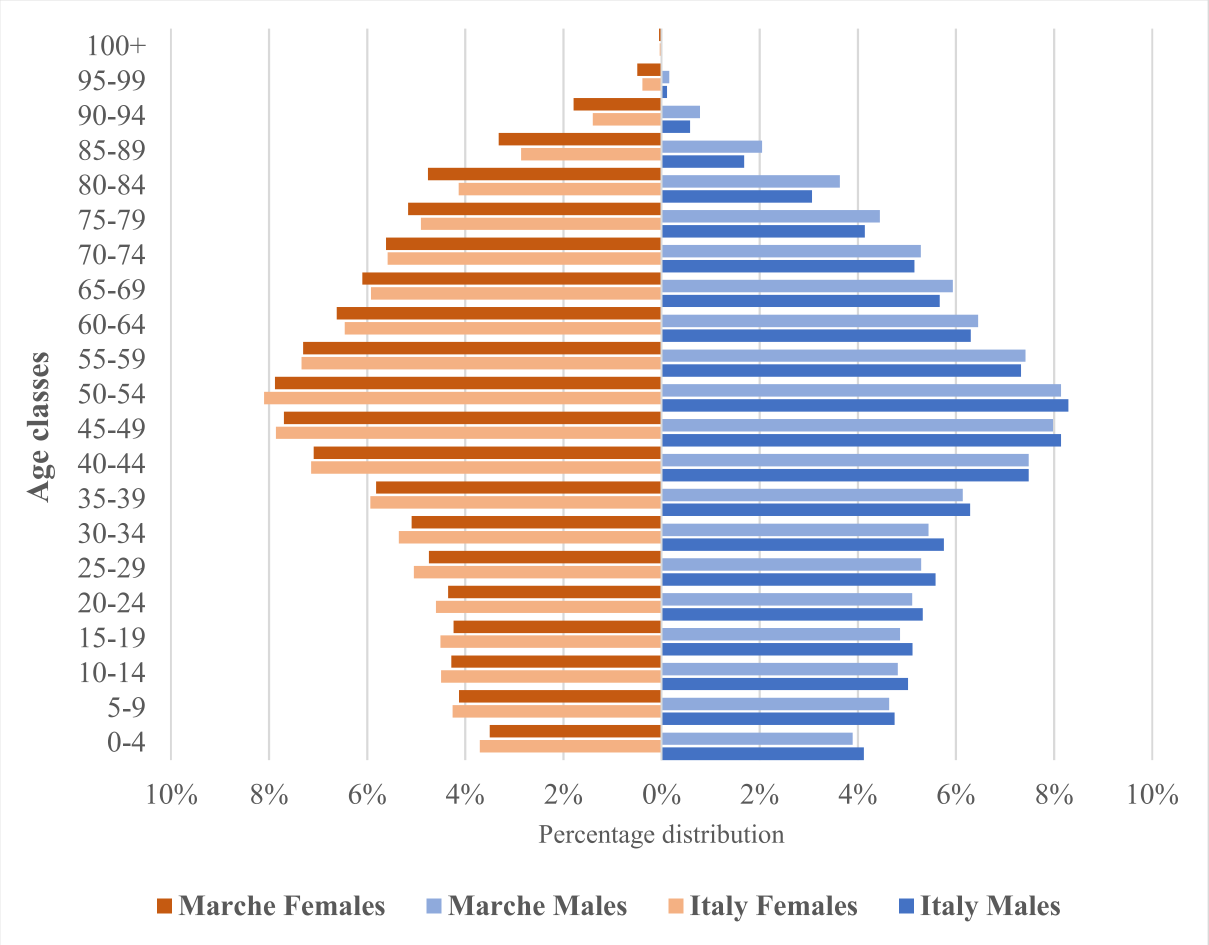

Note. Figure adapted from "I.Stat – Il tuo accesso diretto alla statistica italiana", by the Italian National Institute of Statistics (ISTAT).
